# RNA-Sequencing Analysis Reveals the Role of Mitochondrial Energy Metabolism Alterations and Immune Cell Activation in Form-Deprivation and Lens-Induced Myopia in Mice

**DOI:** 10.3390/genes14122163

**Published:** 2023-11-30

**Authors:** Hojung Kim, Wonmin Lee, Ye-Ah Kim, Sanghyeon Yu, Jisu Jeong, Yueun Choi, Yoonsung Lee, Yong Hwan Park, Min Seok Kang, Man S. Kim, Tae Gi Kim

**Affiliations:** 1 Translational-Transdisciplinary Research Center, Clinical Research Institute, Kyung Hee University Hospital at Gangdong, Kyung Hee University College of Medicine, Seoul 05278, Republic of Korea; kimhj3414@songeui.ac.kr (H.K.); dldnjsals345@khu.ac.kr (W.L.); yeak426@gmail.com (Y.-A.K.); sanghyeon99@khu.ac.kr (S.Y.); symply501@khu.ac.kr (J.J.); uag43@khu.ac.kr (Y.C.); ylee3699@khu.ac.kr (Y.L.); 2 Department of Medicine, Kyung Hee University College of Medicine, Seoul 02453, Republic of Korea; 3 Department of Biomedical Science and Technology, Graduate School, Kyung Hee University, Seoul 02453, Republic of Korea; 4 Department of Microbiology, Ajou University School of Medicine, Suwon 16499, Republic of Korea; parky5@ajou.ac.kr; 5 Department of Ophthalmology, Kyung Hee University Medical Center, Kyung Hee University College of Medicine, Seoul 02447, Republic of Korea; nietzsche@khu.ac.kr; 6 Department of Ophthalmology, Kyung Hee University Hospital at Gangdong, Kyung Hee University College of Medicine, Seoul 05278, Republic of Korea

**Keywords:** myopia, RNA sequencing, animal model, mice

## Abstract

Myopia is a substantial global public health concern primarily linked to the elongation of the axial length of the eyeball. While numerous animal models have been employed to investigate myopia, the specific contributions of genetic factors and the intricate signaling pathways involved remain incompletely understood. In this study, we conducted RNA-seq analysis to explore genes and pathways in two distinct myopia-inducing mouse models: form-deprivation myopia (FDM) and lens-induced myopia (LIM). Comparative analysis with a control group revealed significant differential expression of 2362 genes in FDM and 503 genes in LIM. Gene Set Enrichment Analysis (GSEA) identified a common immune-associated pathway between LIM and FDM, with LIM exhibiting more extensive interactions. Notably, downregulation was observed in OxPhos complex III of FDM and complex IV of LIM. Subunit A of complex I was downregulated in LIM but upregulated in FDM. Additionally, complex V was upregulated in LIM but downregulated in FDM. These findings suggest a connection between alterations in energy metabolism and immune cell activation, shedding light on a novel avenue for understanding myopia’s pathophysiology. Our research underscores the necessity for a comprehensive approach to comprehending myopia development, which integrates insights from energy metabolism, oxidative stress, and immune response pathways.

## 1. Introduction

Myopia, or near-sightedness, is the world’s most common eye disorder and is caused by light focusing in front of the retina, typically caused by axial elongation of the eye during development and into early adulthood [1]. While it is relatively easy to treat with corrective lenses to improve vision, myopia itself is a factor that increases the risk of developing eye diseases such as glaucoma, retinal detachment, and cataracts [2,3]. 

Mechanisms of myopia development have been investigated in animal models; however, the exact mechanism is still unclear [4,5,6]. Representative methods for inducing myopia in animal models include the form-deprivation myopia (FDM) model, which employs a translucent diffuser, and the lens-induced myopia (LIM) model, achieved by imposing defocus through a negative lens causing hyperopic defocus, both of which commonly result in axial elongation [7]. The mechanism underlying this structural change is theorized to be emmetropization, a developmental process that ensures the eye’s optical power matches its axial length, allowing clear focus on the retina. 

Previous studies have identified possible differentially expressed genes and pathways involved in myopia models [8,9,10,11]. However, there is not much of a consensus among the existing studies due to variations in animal models and experimental designs. Additionally, they have uncovered biochemical signaling pathways that translate defocus-related visual stimuli into retinal cellular and biochemical changes, which then signal changes in the retinal pigment epithelium (RPE), choroid, and ultimately sclera, resulting in altered eye growth and refractive state changes. However, the differences between FDM and LIM models have not been clearly reported.

There may be a difference between the FDM model and the LIM model in the mechanism that causes myopia. Research conducted on chicks has revealed that the dopamine mechanisms responsible for mediating the protective effects of brief periods of unrestricted vision might vary between the FDM and LIM models [12]. This suggests a potential divergence in the mechanisms governing growth control between these two methods. However, to our best knowledge, there are limited reports about the differences between these two forms of myopia models with the same experimental conditions, such as experimental duration and environment.

In the present study, we compare FDM and LIM in mice under the same experimental conditions to identify common genes and pathways involved in the development of myopia differential expression analysis and pathway analysis with mice RNA data from these two myopia models and a control.

## 2. Materials and Methods

### 2.1. Animals

**Figure 1:**
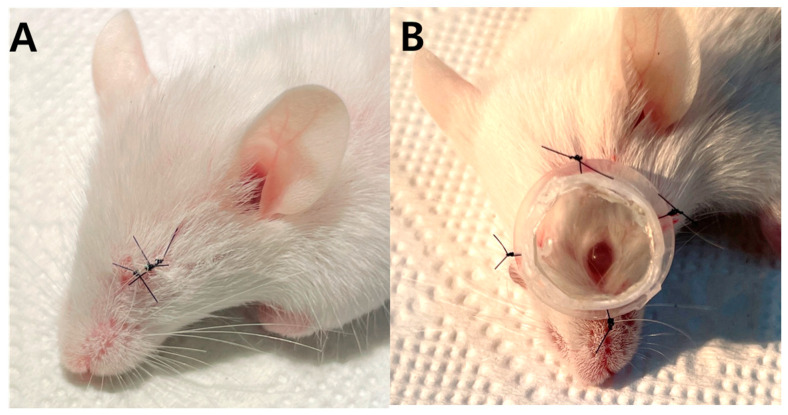
Form-deprivation myopia (**A**) and lens-induced myopia (**B**) in mice.

All procedures adhered to the Association for Research in Vision and Ophthalmology (ARVO) statement on the use of animals in ophthalmic and vision research and were approved by the animal institutional review board of Kyung Hee University hospital at Gangdong (KHNMC AP 2022-014). Nine male three-week-old Balb/c mice weighing 10–15 g were used in this study. Three mice were assigned to each experimental group (three for the control, three for LIM, three for FDM). After being anesthetized via an intramuscular injection of alpaxalone (10 mg/mL, Alfaxan, Jurox, Kansas City, MO, USA), in the LIM group, a −10.0 diopter (D) lens was affixed to a frame created through the processing of an Eppendorf tube. This frame was subsequently secured onto the left eye utilizing 8-0 prolene sutures on animal skin (Figure 1). In the FDM group, the left eye was sutured using 8-0 prolene. The control group did not receive any form of treatment. After four weeks, the animals were euthanized using a CO_2_ chamber and enucleated. Total RNA was extracted from their whole eyes.

### 2.2. RNA-Seq Preprocessing

Raw datasets (i.e., fastq) of nine samples in total, where three were from the LIM model, three from the FDM model, and the other three from the control group, were acquired from bulk RNA sequencing. Through the sequencing process, paired-end reads whose length was 101 bp were generated for all the samples using the illumina platform with the truseq stranded mRNA library prep kit. Next, we applied a preprocessing procedure; (i) alignment: STAR aligner (v2.7.3a) was executed [13]; (ii) quantification: HTSeq-count (v0.12.4) was implemented [14]; (iii) normalization: VST (Variance Stabilizing Transformation) was applied using the DESeq2 package [15]. The reference genome, GRCm39, with its annotation was utilized in the mapping for all the reads of the three conditions.

### 2.3. Differential and Enrichment Analysis

We performed two different gene expression profile comparisons such as ‘LIM vs. control’ and ‘FDM vs. control’ using the DESeq2 package (*p* < 0.05) whose visualization was implemented using EnhancedVolcano [16] and clusterProfiler [17]. Four different gene sets were used for the enrichment analysis with gene ontology terms; (i) all aligned genes, (ii) genes only associated with eyes, (iii) genes only associated with the immune system, and (iv) genes only associated with inflammation. These gene groups were selectively listed using the Molecular signature database (MSigDB) with corresponding keywords [18,19,20]. Pathway enrichment analysis through Gene Set Enrichment Analysis (GSEA) was executed and displayed using Cytoscape [21] along with its visualization app EnrichmentMap [22]. From the DEGs, Protein–Protein Interaction (PPI) networks were produced, where each interaction score was higher than 0.9, through STRING [23], which is a protein interaction database including known direct or indirect associations. Another pathway enrichment analysis was conducted through Fast Gene Set Enrichment Analysis (fgsea) [24] utilizing DEGs of MitoCarta 3.0 [25]. In these analyses, five genes (Alas2, Hbb-bs, Hbb-bt, Hba-a1, Hba-a2) which are marker genes of red blood cells were removed from the downstream analysis to consider possible contamination with blood.

### 2.4. Analysis on Mitochondria-Associated Expression Profiles

The custom-made gene list of mitochondrial regulatory mechanisms in [26] was used to selectively explore gene expression profiles associated with mitochondrial energy metabolism across mitochondria-associated pathways. Gene lists of mitochondrial complexes and additional lists of energy metabolism-associated pathways were applied. To visualize gene regulations involved in the KEGG pathway of “oxidative phosphorylation”, the Pathview package of R was used [27].

## 3. Results

### 3.1. Eye-Associated Differentially Expressed Genes between LIM and FDM Are Different

**Figure 2:**
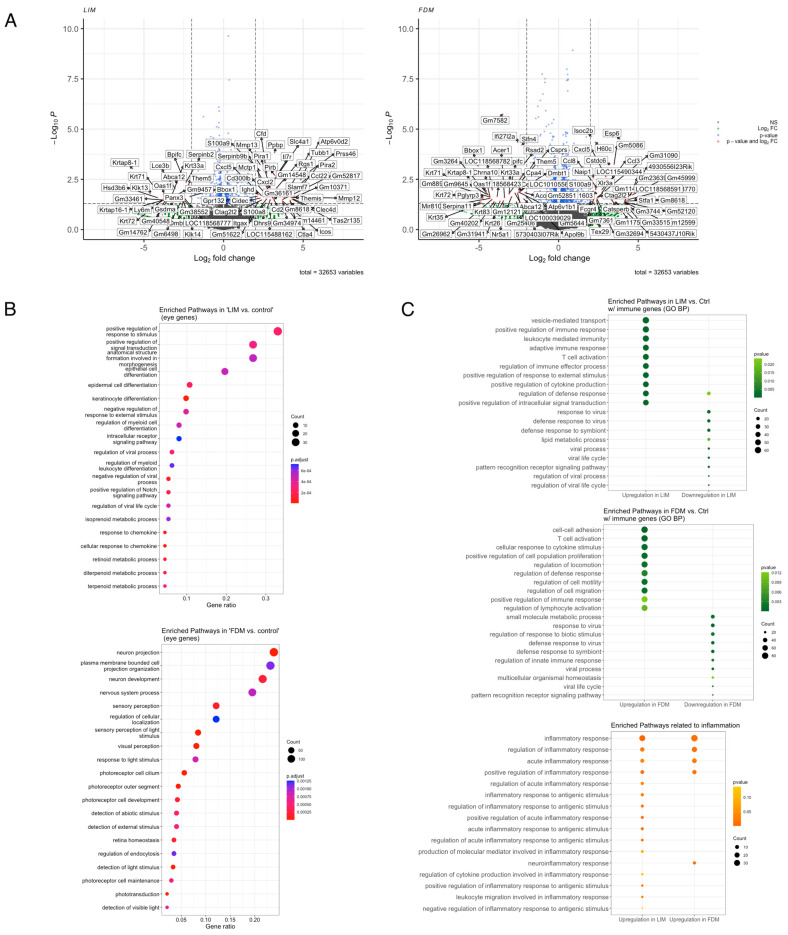
Differentially expressed genes and enriched pathways. (**A**) Differentially expressed genes in LIM and FDM groups compared to the control group (*p*-value < 0.05 and log2 fold-change > 2). (**B**) Top 20 most enriched pathways of known eye-associated genes from MSigDB in LIM and FDM compared with the control (*p*-value < 0.1 and q-value < 0.25). (**C**) Top 10 most enriched pathways directly associated with immune and inflammation in LIM and FDM compared with the control (*p*-value < 0.1 and q-value < 0.25).

To explore the functional association of transcriptional profiles of LIM and FDM, we first compared them with the control and identified 503 genes from LIM and 2362 genes from FDM that were significantly differentially expressed. As shown in Figure 2A, some overlapping differentially expressed genes (DEGs) including Ctag2l2, Gm8618, S100a9, and Cxcl5 exhibited upregulation in both LIM and FDM, while Krtap8-1, Oas1f, Krt71, Krt331, Krt72, Dmbt1, Them5, Bpifc, and Abca12 were commonly downregulated in both of them. Among those genes, Ctag2l2, whose human ortholog, CTA1A, was one of the human myopia candidate genes [28] and S100a9, known to be implicated in the regulation of the immune response, was previously identified to be upregulated in a guinea pig FDM model [10]. We further inquired into eye-specific functional associations by performing Gene ontology (GO) analysis using only eye-associated genes obtained from MSigDB (Figure 2B). While signaling pathways such as response to stimulus, signal transduction, structure formation in morphogenesis, and epithelial cell differentiation were listed as the top four significantly changed pathways in LIM, FDM exhibited significant alterations on pathways such as neuron projection, plasma membrane bounded cell projection organization, neuron development, and nervous system process at the top.

Other pathways, however, were found to be directly or indirectly associated with immunologic pathways in both LIM and FDM, while upregulation of immune-associated GO terms has been observed in previous studies [29,30] and the importance of inflammation in myopia progression has been proven clinically and experimentally [31]. To further look into this association, we performed GO analysis using only either immune-associated or inflammation-associated genes selected from MSigDB. T cell activation, regulation of defense response, and positive regulation of immune response were revealed as commonly upregulated in both LIM and FDM. However, response to virus, defense response to symbiont, viral process, viral life cycle, and pattern recognition receptor signaling pathway were shown in common downregulation between LIM and FDM. For inflammation-specific pathways, while more inflammatory pathways were enriched in LIM, (positive) regulation of inflammatory response and acute inflammatory response were commonly listed up in both LIM and FDM.

### 3.2. GSEA & PPI-Based Clusters Exhibited Significant Alterations in Immune-Associated and Energy Metabolism

**Figure 3:**
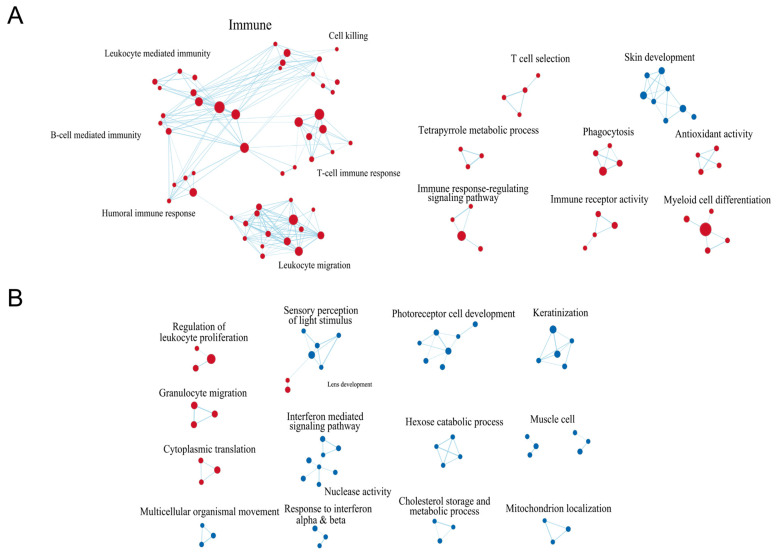
GSEA-based network visualization in LIM and FDM groups compared to the control group. (**A**) Network association of LIM compared with the control. (**B**) Network association of FDM compared with the control. Edges are inferred by considering strong correlation between signaling pathways and each node (as enriched pathway) size corresponds with number of edges. The color is proportional to degree of differentiation, where up/downregulation indicates red or blue, respectively. The selected pathways were filtered using false discovery rate (FDR) < 0.75 and edge similarities > 0.375.

To determine the underlying biological mechanisms of myopia, we implemented Gene Set Enrichment Analysis (GSEA) to further explore interactions between pathways as illustrated in Figure 3A,B. Primarily overlapping pathway clusters between LIM and FDM turned out to be immune-associated clusters, where LIM exhibited more extensive interactions encompassing not only lymphocyte regulations, such as regulation of B or T cells, whereas myeloid cell regulations such as granulocyte migration, granulocyte chemotaxis, and neutrophil migration. The humoral immune response featured multiple interactions with B and T cell responses, suggesting that its complement system might be involved in modulating the immune response in myopia, as previous studies presented that patients with myopia have an elevation of complement components in their serum and aqueous humor [32,33,34]. LIM also disclosed LIM-specific immune-associated clusters including T cell selection, immune response regulating signaling pathway, immune receptor activity, phagocytosis, and myeloid cell differentiation. Only one cluster, skin development, comprising intermediate filament, keratin filament, keratinocyte differentiation, and keratinization was in downregulation in LIM. Several clusters that could be possibly associated with eye development such as photoreceptor cell development, sensory perception of light stimulus, and mitochondrial localization were suppressed in FDM. Specifically, the suppression of mitochondrial energy metabolism along with the activation complement system aligns with the findings of previous studies on myopia development [35,36,37].

**Figure 4:**
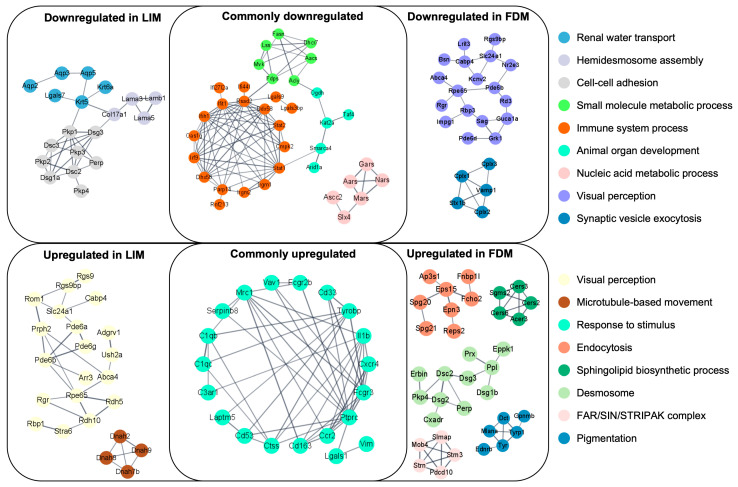
Protein–Protein Interaction (PPI) networks based on differentially expressed genes (DEGs) in LIM and FDM groups compared to the control group. Each node represents a protein mapped to one of the DEGs, and the edges represent strong interactions between corresponding proteins. DEGs were selected through *p*-values of 0.2 for LIM and 0.1 for FDM. Interactions with protein interaction scores higher than 0.9 were included into clusters where each cluster contained at least five proteins.

To explore other potential functional associations, we searched for any potential clusters on the Protein–Protein Interaction (PPI) network between LIM and FDM using STRING and visualized them using Cytoscape. The primary revelation from this PPI-based analysis was metabolic processes in downregulation such as the small molecule metabolic process or the nucleic acid metabolic process shown in Figure 4 as commonly downregulated. This observation is in line with the aforementioned finding in downregulation through GSEA since metabolic processes are associated with mitochondrial energy metabolism. Other commonly up/downregulated clusters turned out to be at partially at least associated with pathways of immune processes as already demonstrated in significant alterations analyzed through GSEA. Additionally, myopia-model-specific up/downregulation included protein interactions of protein/cell adhesion such as hemidesmosome assembly or cell–cell adhesion in downregulation, and visual perception in upregulation for LIM. Also, protein interactions of visual perception in downregulation and membrane-associated transport or cellular processes such as endocytosis or desmosome in upregulation for FDM were represented. The different alteration pattern of visual perception could be interpreted as a compensatory mechanism using different sets of genes. Considering the downregulations of mitochondrial energy metabolism and some metabolic processes through GSEA and PPI-based analyses might suggest the probable direct/indirect association between a broad spectrum of alterations in energy metabolism and myopia development.

### 3.3. Some Energy-Associated Metabolism Pathways Demonstrated Significant Alterations

**Figure 5:**
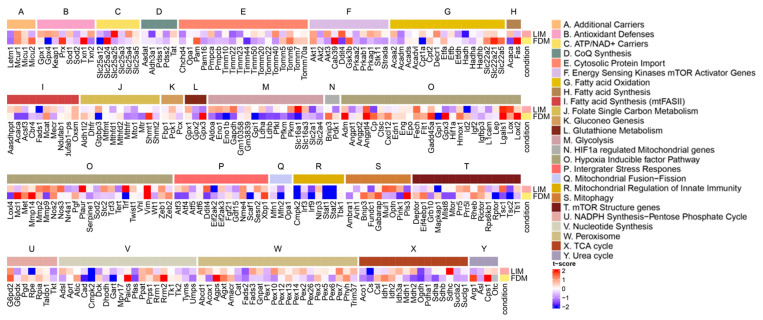
Heatmap of pathways associated with mitochondrial energy metabolism. Transcriptional profile alterations (as t-scores) of the customized mitochondria-associated gene set obtained from [26] were visualized. Red indicates upregulation in either the LIM or FDM compared to the control, whereas blue indicates downregulation in either the LIM or FDM compared to the control.

Since the retina is one of the most metabolically active tissues [38], we explored transcription profiles of pathways directly or indirectly associated with energy metabolism to compare relative expression levels of specific genes of the pathways (Figure 5). For essential pathways of energy metabolism, while fatty acid oxidation did not divulge considerable alterations, the TCA cycle disclosed slight up/down variations across its associated genes. According to studies from animal models or human patients [39,40], the TCA cycle was found to be one of the most impactful pathways as downregulated on myopia, while Cs and Ogdh could be potential target genes based on our analyses. Another major pathway, glycolysis, revealed significant alterations, but differently between LIM and FDM. This upregulation of glycolysis on LIM could be interpreted as upregulated glycolytic enzymes and pathways in LIM, as supported by a proteomics study using chicks [41]. FDM suppression could also be considered, as a metabolomics study utilizing guinea pigs reported that glucose was found to accumulate in the form-deprived eyes, suggesting a decrease in aerobic glycolysis [40]. In addition, previous studies discovered that a myopic retina experiences oxidative stress due to its hypoxic conditions given from the retina’s high blood flow and susceptibility to photic oxidative injury [42]. We looked over pathways associated with oxidative stress such as NADPH synthesis, integrator stress response, and antioxidant defenses. Based on the gene expression patterns of those pathways, G6pd2 and Rpia in NADPH synthesis and Nme4 and Xbp1 in integrator stress response might be potential candidate genes of oxidative stress on myopia.

### 3.4. Oxidative Phosphorylation Revealed Specific Alterations

**Figure 6:**
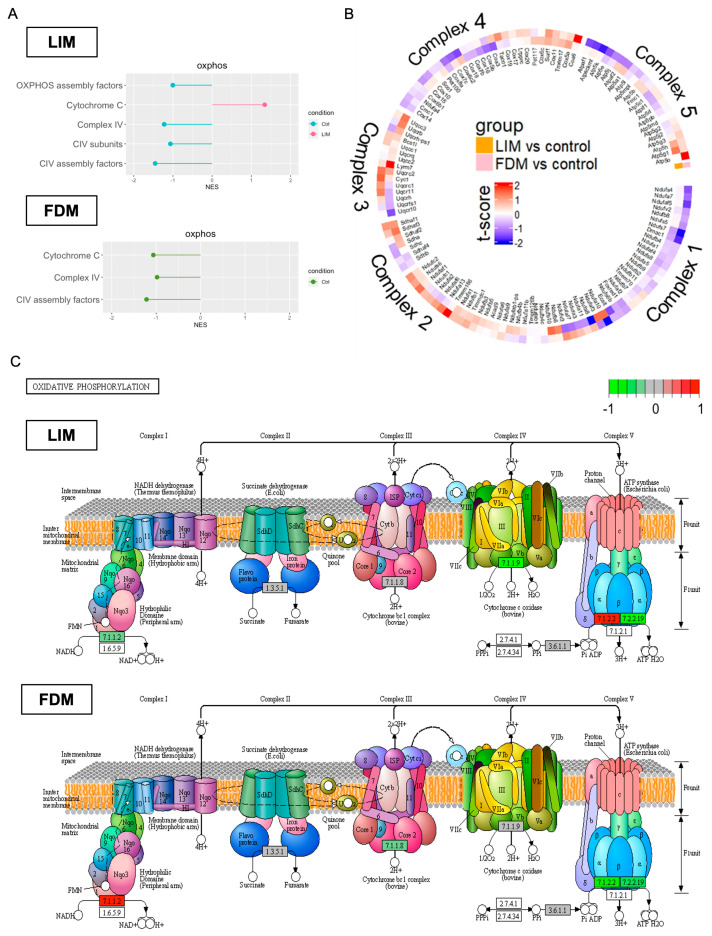
Multiple comparative analyses of Oxidative Phosphorylation (OxPhos) in LIM and FDM in comparison to the control group. (**A**) Up/Downregulation of OxPhos-associated pathways through GSEA (fgsea). Common gene lists between DEGs (*p*-value < 0.5) and MitoCarta 3.0 were used. (**B**) Transcriptional profile alteration of OxPhos with t-score. (**C**) OxPhos complex-specific gene expression changes using Pathview [27]. All the genes used for the three different analyses were DEGs with *p*-value < 0.5.

To further investigate changes in energy metabolism, we looked into oxidative phosphorylation (OxPhos), the most crucial functional process for energy production, through three different analytic views. While OxPhos was generally downregulated in both LIM and FDM, common/different alterations across five distinct complexes were taken into consideration. The first analysis through fgsea with MitoCarta 3.0 (Figure 6A) displayed common suppression on OxPhos complex IV in both LIM and FDM. Cytochrome C was, however, upregulated in LIM while downregulated in FDM. In a previous study with an FDM model of guinea pigs, both protein and mRNA expression levels of cytochrome C protein (SCO1 and SCO2) were found to be considerably lower in the FDM group [43]. The second analysis in Figure 6B represented relative expression levels of all five complex genes. The circular heatmap showed common downregulation on complex I and IV in both LIM and FDM, but different regulation on complex V by relative downregulation in FDM. The third analysis (Figure 6C) provided KEGG-based visualization of complex-associated gene expression levels. While downregulation occurred in complex III of FDM and complex IV of LIM, subunit A of complex I (NADH ubiquinone oxidoreductase) was downregulated in LIM while it was upregulated in FDM. Also, in complex V, F-type H+ transporting ATPase subunit α was upregulated in LIM while downregulated in FDM, and H+/K+ exchanging ATPase subunit α was downregulated in both LIM and FDM.

### 3.5. Metabolic Alterations Occurred Extensively

**Figure 7:**
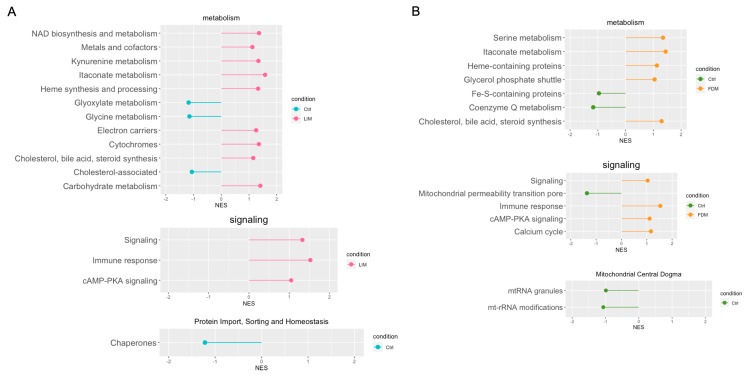
Lollipop based on GSEA with fgsea using MitoCarta3.0 in LIM and FDM groups compared to the control group. (**A**) Enriched pathways in LIM on metabolism/signaling/protein import, sorting and homeostasis. (**B**) Enriched pathways in FDM on metabolism/signaling/mitochondrial central dogma. Common gene lists between DEGs (*p*-value < 0.5) and MitoCarta 3.0 were used.

In the pursuit of prediction of metabolic alterations, we proceeded with a further GSEA analysis implemented through fgsea with MitoCarta 3.0 on metabolic alterations of either LIM or FDM extensively (Figure 7). For energy-associated metabolism other than OxPhos changes, pathways of LIM (NAD biosynthesis and metabolism, electron carriers, and carbohydrate metabolism) and coenzyme Q metabolism of FDM displayed significant alterations. Additionally, in FDM, mitochondrial permeability transition pore demonstrated sufficient change, suggesting disruption of mitochondrial calcium homeostasis from induction of myopia [44]. Several other significant alterations of metabolic pathways that were not directly associated with energy metabolism were divulged, where commonly more activated metabolisms in both LIM and FDM turned out to be not only lipid-associated metabolism, but also immune-associated metabolism. For lipid metabolism, cholesterol, bile acid, and steroid synthesis were upregulated for both, as supported by previous studies which show enrichment of bile secretion and its associated pathways in FDM of chick models and human myopia patients [36]. Immune-associated metabolism such as immune response, cAMP-PKA signaling, and itaconate metabolism disclosed relatively further activations in both LIM and FDM. cAMP is the core regulator of both pro/anti-inflammatory activities along with numerous other physiological processes [44] and commonly upregulated pathways also included itaconate metabolism, which is known to play an essential role in immunity or inflammation [45]. Additionally, LIM showed downregulation of chaperones in protein-related pathways which could imply decreased activation to deal with stress conditions during myopia induction. In FDM, mitochondrial central dogma with mtRNA granules and mt-rRNA modifications was downregulated. Modifications of mitochondrial rRNA ensure the stability of mitochondrial protein translation and dysregulation of such modifications may lead to fatal mitochondrial and metabolic dysfunction [46]. Therefore, although different pathways showed alterations, disruption of mitochondrial protein homeostasis was present in both LIM and FDM.

## 4. Discussion

In this study on myopia, we juxtaposed both the FDM and LIM mouse models by employing comparable experimental settings to examine not only commonly associated, whereas model-specific regulatory mechanisms in myopia development. This investigation explored differential transcriptional profiles and their associated pathway activities by applying all gene lists, known eye-associated gene lists, and specific gene lists of energy metabolism-associated pathways.

The significance of immune responses in the progression of myopia has been well-documented both clinically and experimentally [31] and several studies have already noted the upregulation of immune-associated GO terms [47]. Our GO assessment of DEGs also revealed an increased activation of immune-associated pathways in both LIM and FDM. When the analysis narrowed down to genes specifically tied to the immune system, both LIM and FDM frequently showed upregulation in pathways associated with inflammation and its positive regulation. Particularly, LIM displayed a more pronounced activation of pathways linked to immunity compared to FDM. These pathways spanned responses managed by T cells, as well as those linked to B cells and the humoral immune system. Many genes related to the immune system have been known to be involved in the development of myopia [10], for instance, CTLA4 from our analysis involving T cell regulation was found to be uniquely upregulated in LIM, supporting a surge in the expression of several LIM-specific lymphocyte immune pathways. Of significant note, the humoral immune system saw extensive activation specifically within the complement system while interacting directly with B and T cell-driven responses, where multiple research endeavors have delved into the influence of the complement system on myopia [48]. As depicted in Figure 4 as protein–protein interaction-based clusters, the centrality of immune responses in pathway evaluations and their interplay was further emphasized at the proteomic level. These interactions were involved in systems such as chemokine receptors, binding of chemokine, the general immune framework, and the innate immunity system. 

While many previous studies have primarily focused on the retina or posterior parts of myopia, our study analyzed the whole eyes [49,50]. Although this approach might pose challenges for a detailed examination of individual ocular structures, it has enabled us to view a wide array of pathways associated with eye development during the myopia induction process simultaneously. Considering pathways directly/indirectly associated with visual perception, some discrepancies between LIM and FDM have been captured. In the FDM model, several pathways associated with visual perception were observed in downregulation and the reduction in light stimulus perception seemed similar to the form-deprivation technique used for inducing myopia. This combinatorial downregulation of the nervous system process could not only substantiate the modified visual perception inherent to myopia, it could also emphasize the significant role of contrast sensitivity in refractive eye development of myopia since they were reported to be associated with contrast sensitivity in the FDM model of myopia [51]. LIM model clusters from the PPI network, however, stood in sharp contrast to GO analysis of the FDM model for the alterations of visual perception. This corroborates observations from the guinea pig LIM model, where phototransduction was highlighted as one of the most significant pathways [52]. In addition, pathways associated with epithelial development exhibited contrasting behaviors: downregulation in LIM and upregulation in FDM. The modulation of epithelial development of the LIM model, particularly the regulation of water loss via the epithelium, suggested a potential disruption in water homeostasis within the eyes. This phenomenon aligns with one of the primary mechanisms implicated in myopia development [29]. The observed downregulation of epithelial cells was also believed to reflect the alterations in soft tissues during the axial lengthening process, since changes in epithelium thickness in myopia are known to decrease in the central cornea and the central epithelium is linked with refractive errors [53]. Therefore, the downregulation of the epithelial cell pathway during myopia induction likely mirrors changes associated with an increase in eye axial length, as well as alterations in refractive change.

This study stands out by shedding light on the comprehensive changes in energy metabolism associated with myopia, extending beyond the insights provided by earlier research. One of the intriguing findings is the notable alterations in oxidative phosphorylation. These alterations, taken in conjunction with the broader shifts in energy metabolism, signal a potential ocular response to hypoxic damage. Hypoxia, or reduced oxygen supply, can prompt a cascade of metabolic and cellular reactions, including adaptations in energy production mechanisms. Several pathways closely linked to the electron transport chain were notably enriched in our myopia samples, indicating changes in the energy metabolism of eye development, as in previous research which has drawn attention to modifications in oxidative phosphorylation and their potential roles in myopia progression. A previous study using the LIM chick model found a significant portion of core genes in upregulated mitochondrial metabolism pathways to form part of complex I, III, and IV with additional 11 core genes from other pathways coding for complex V [37]. Another study demonstrated that a variant of the NDUFAF7 gene that might contribute to the development of pathological myopia by decreasing complex I activity resulted in decreased ATP and GTP production downstream, which weakened photoreceptor cell function [54]. A meta-analysis of transcriptome data reported upregulation of the NDUFS5 gene in late stage of myopia, which encoded a subunit of complex I [48]. Our results show how metabolic changes in LIM and FDM may differ from each other in the electron transport chain pathway. Oxidative phosphorylation in this study was predominantly downregulated in both LIM and FDM, albeit with slight variations; (i) cytochrome C: upregulation in LIM, but downregulation in FDM; (ii) subunit A of complex I: downregulation in LIM, but upregulation in FDM; (iii) complex IV: unique downregulation in FDM; (iv) complex III: unique downregulation in LIM. Other than OxPhos, the FDM model showed a suppression in the TCA cycle, while the LIM model exhibited broader metabolic changes including increased carbohydrate metabolism and decreased glyoxylate and glycine metabolism. Several metabolomic studies on animals and humans have identified the TCA cycle as a key pathway as downregulated in myopia [39], which might be associated with axial length elongation [55]. Furthermore, considering high metabolic activities of the retina, neutralization of reactive oxygen species that have an effect on hypoxia in myopia development might have a significant role in myopia development since oxidative damage to the retina is commonly associated with hypoxic conditions [56].

One major limitation of this study comes from its experimental design. The ocular growth is thought to be controlled locally by the posterior eye, and therefore, most myopia studies have analyzed RNA extracted after isolating posterior eye structures including the retina and the choroid. However, the RNA was extracted from the whole eye in this study and this may have resulted in less sensitive results.

## 5. Conclusions

The association between energy metabolism alterations and immune cell activation might open up a new avenue for understanding the pathophysiology of myopia. The immune system’s role in myopia development has been a topic of growing interest, and our findings hint at a deeper interplay between metabolic changes and immune responses. It is plausible that as energy metabolism shifts, it could trigger certain immune responses, either as a direct result of metabolic byproducts or as a broader adaptive mechanism to maintain ocular homeostasis. Overall, our research underscores the need for a more comprehensive approach to understand myopia development, integrating insights from energy metabolism, oxidative stress, and immune response pathways. Such an integrated perspective could pave the way for more targeted therapeutic interventions in the future.

## Data Availability

The data presented in this study are available on request from the corresponding author.
